# Oxalic Acid in Combination with Iron and Mangnese Peptonates

**Published:** 1894-12

**Authors:** 


OXALIC ACID IN COMBINATION WITH IRON AND MANGANESE PEPTONATES, AS AN EMMENAGOGUE IN CHLOROSIS. (*Medical News*, Sept. 29, 1894.) By H. C. Bloom, M. D.—This is a second report by Dr. Bloom, on the value of oxalic acid as an emmenagogue. Since his previous paper, he has used the drug in upward of one hundred cases of amenorrhœa, and, while it occasionally failed him, his experience still led him to regard it as the surest and safest emmenagogue we have. He thought it also reliable, under certain conditions, as an oxytocic. In chlorosis, additional therapeutic measures were necessary, looking towards the establishment of a better condition in the blood. Frequently, however, even when the anæmia disappears, the amenorrhœa may persist. In such cases the combination of the ferruginous preparation with oxalic acid has yielded, in his experience, the best results. His formula is as follows:

℞. Ferri peptonat, gr. xii.
Mangani peptonat, gr. ii.
Acid, oxalic, C. P., gr. ii.
Alcoholis, ʒiii.
Aquæ, q. s. ad. ℥iv. M.
Sig.—Two drachms *ter in die.* —*Ex.*

